# Organized Atrial Tachycardias after Atrial Fibrillation Ablation

**DOI:** 10.4061/2011/957538

**Published:** 2011-09-19

**Authors:** Sergio Castrejón-Castrejón, Marta Ortega, Armando Pérez-Silva, David Doiny, Alejandro Estrada, David Filgueiras, José L. López-Sendón, José L. Merino

**Affiliations:** ^1^Robotic Cardiac Electrophysiology Unit, Department of Cardiology, University Hospital La Paz, Paseo de la castellana, No 261, 28046 Madrid, Spain; ^2^Department of Pediatric Cardiology, University Hospital La Paz, Paseo de la castellana, No 261, 28046 Madrid, Spain

## Abstract

The efficacy of catheter-based ablation techniques to treat atrial fibrillation is limited not only by recurrences of this arrhythmia but also, and not less importantly, by new-onset organized atrial tachycardias. The incidence of such tachycardias depends on the type and duration of the baseline atrial fibrillation and specially on the ablation technique which was used during the index procedure. It has been repeatedly reported that the more extensive the left atrial surface ablated, the higher the incidence of organized atrial tachycardias. The exact origin of the pathologic substrate of these trachycardias is not fully understood and may result from the interaction between preexistent regions with abnormal electrical properties and the new ones resultant from radiofrequency delivery. From a clinical point of view these atrial tachycardias tend to remit after a variable time but in some cases are responsible for significant symptoms. A precise knowledge of the most frequent types of these arrhythmias, of their mechanisms and components is necessary for a thorough electrophysiologic characterization if a new ablation procedure is required.

## 1. Introduction

Organized atrial tachycardias (AT) are a common problem after atrial fibrillation (AF) ablation (post-AF ablation AT—PAFAT). Since the first isolated case reports [1–5], several mechanisms [6] and different times of onset following the index procedure have been reported.A new ablation procedure often solves this arrhythmic problem [5, 7–14]. Nevertheless, this rhythm disorder merits a special attention for different reasons: (1) it has a high incidence and is often very symptomatic, (2) the complexity of the atrial arrhythmogenic substrate, which may be responsible for the frequent concurrence of several types of AT mechanisms in the same patient, (3) the variety of mapping and ablation approaches which have been reported and (4) the fact that PAFATs mechanisms may be linked to the mechanisms responsible for AF maintenance.

This paper reviews the incidence, clinical presentation, mechanisms, electrophysiological characterization and ablation of PAFAT. Finally, a brief review of organized ATs presenting during the AF ablation procedure is also provided.

## 2. Incidence

The real incidence of PAFAT cannot be easily extracted from published series because most of them either focused just on left [15–17] or macroreentrant (MR) AT [18, 19], or did not report the incidence of cavotricuspid isthmus-dependent (CTI) atrial flutter, which is responsible for 7–10% [20–22] of all PAFATs. This latter figure is even higher in patients with previous cardiac surgery [23]. In addition, the reported incidence of PAFAT is probably underestimated in most of these series because only symptomatic patients were referred for a new ablation procedure [21, 24]. All these reasons explain in part that the reported incidence of sustained PAFAT varies widely. However, differences in PAFAT incidence mainly depend on the following two factors: the predominant type of AF before the index procedure and the approach [8, 12, 13] used for AF ablation. 

The incidence of PAFAT ranges between 4.7 and 31% and is usually higher after circumferential pulmonary veins (PV) ablation using wide-area circular lesions around ipsilateral PVs (*circumferential pulmonary vein ablation*—CPVA) [25] or when additional ablation lines are incorporated in the procedure than with other ablation approaches [15–18, 20, 21, 24, 26, 27]. This group of patients whose AF was treated with CPVA is by far the one in which PAFATs have been most extensively studied. However, several variants of the original CPVA technique have been reported in recent years. Abatement of PV electrograms within the encircled area was the main endpoint of the original series and PV electrical isolation (PVI) has been required only in the most recent ones [26–28]. Despite this evolution [29], the PAFAT incidence apparently has remained apparently unchanged. The number and location of additional ablation lines had been also heterogeneus among the different reported series but it seems that additional lines may have a greater impact on PAFAT incidence than on AF recurrences. Pappone et al. [25] and Anousheh et al. [27] found that by adding roof, posterior, or mitral isthmus ablation lines the development of new-onset macroreentrant AT was reduced in comparison to CPVA alone, as far as conduction block across these lines was achieved. 

The incidence of AT is much lower after less extensive AF ablation approaches such as segmental ostial PVI [1, 30, 31] or circumferential antral PVI [32], especially when no additional ablation lines are used or only the electrically active PVs are targeted [23, 24, 33–39]. The incidence of AT with these latter approaches ranges between 2% and 7.7%. The only discrepant incidence value with this approach is the 29% found by Ouyang et al. [38] but that one could have been related to the modification of the standard PV isolation technique which these investigators used. The lower incidence of PAFAT found following segmental ostial PVI could be partially related to the smaller proportion of patients enrolled with persistent/chronic AF enrolled in these studies, as opposed to the studies in which CPVA was employed. Patients with long-standing persistent/chronic AF have both more electrical and anatomical atrial remodeling [40, 41] and low-voltage and scar areas [34] than patients with paroxysmal AF. These scar areas have been associated with MR circuits and may configure the substrate for organized AT [13, 34, 35]. This hypothesis was congruent with a ninefold higher AT incidence in patients with persistent forms of AF reported by Porter et al. (2,4% versus 20%) [42].Actually, this is not the case because the proportion of patients with persistent/chronic AF in the studies in which CPVA plus lines was used reached 17%–36,5% [15–18, 20, 21, 24, 26, 27], a percentage similar to that reported in the series in which segmental or circumferential antral PV isolation was performed (8%–43%) [23, 24, 33–39]. As a consequence, factors directly linked to the ablation technique in itself seem to determine the PAFAT incidence, although the scarce studies that have compared both techniques have yielded conflictive results [43, 44].

Finally, there are some other strategies globally characterized by an extensive atrial ablation which aims at terminating AF, rendering it noninducible or at least transforming it into an organized AT amenable to mapping: complex fractionated atrial electrograms (CFAEs) ablation [45], alone or as an adjunct to PVI isolation, and stepwise approaches comprising sequential addition of conventional techniques [46]. The incidence of PAFAT in patients initially treated with CFAE ablation alone or combined with PVI [45, 47–53] is 7,6%–24%, but Nademanee et al. published a remarkable study with 674 patients in which a low incidence of right atrial flutter (2,4%) and no cases left AT [52] were reported. Most of the patients included in these series suffered persistent, permanent, or chronic AF (33%–100%). On the other hand, a number of papers have reported the incidence of AT after stepwise AF ablation or addressed specifically AT appearing after these approaches [46, 53–59]; the incidence of PAFAT in this context oscillates between 23%–44%. Stepwise techniques are resorted to for long-lasting persistent forms of AF almost exclusively (23%–100% of patients in these series).

## 3. Clinical Aspects

From a clinical point of view, PAFATs are characterized by: early onset after the index procedure, multiplicity of arrhythmia types in the same patient (not always recognizable on the surface ECG), frequent and important symptoms refractory to management with rate-controlling drugs, limited amenability with antiarrhythmic drugs, high recurrence rate after cardioversion, and to sum up, very frequent requirement of at least one subsequent ablation procedure to cure them.

### 3.1. AT Time of Onset after AF Ablation

Chang et al. [28] demonstrated that multiple ATs can be induced in 16,3% of patients immediately after circumferential PVI isolation, and this proportion rises (38%) when a more extensive ablation is used [19]. In other cases, an organized AT of a “totally” different nature such as typical CTI-dependent flutter appears during AF ablation [60]. These findings suggest that the substrate capable of maintaining organized ATs is already present at this stage. It may explain why the onset of AT occurs relatively early in the follow-up after AF ablation, with an average time of onset between 2, 7–13 weeks [15–19, 55]. Ouyang et al. [38] and Themistoklakis et al. [35] reported that more than 80% of the ATs they studied had appeared during the first 2 and 4 weeks after the AF ablation procedure, respectively.

### 3.2. Clinical Course

Chugh et al. [18] proposed to reserve a new ablation for patients in whom the AT eventually persists symptomatic after a prudential observation period, because according to their reported experience, up to a third of patients presented complete resolutions of their ATs (spontaneously or after electrical cardioversion) and an additional 4.7% achieved good clinical control under pharmacologic treatment alone. This cautious proceed is further guaranteed because in other studies, with different AF ablation techniques, 45–64% of patients remained free of AT recurrences after a few months had elapsed [15, 17, 19, 45]. 

Although PAFATs are most commonly persistent (78% [59]–92% [56]) instead of paroxysmal, they tend to cause important symptoms (even syncope in 28% of patients studied by Pappone et al. [17]). Commonly, PAFAT are more problematic for patients than the original AF, specially because organized atrial arrhythmias are usually associated to faster ventricular response thus requiring electrical cardioversion more frequently [15, 19, 20]. In addition, these PAFATs respond poorly to antiarrthythmic drugs (only 4.7% [22]–18% [20] of patients on antiarrhytmics are oligosymptomatic enough as to reject a new ablation procedure), and in some cases interruption of antiarrhythmics could be beneficial to avoid recurrences [33].

### 3.3. Multiple Different Arrhythmias in the Same Patient

This is one of the most prominent features of PAFAT. Haïssaguerre et al. [56] and Deisenhofer et al. [15] described 70–81% of their patients to have more than one arrhythmia mechanism during the electrophysiologic study. Other authors [15, 19, 57, 58, 61] have reported that the average number of different AT mechanisms per patient varies between 1.8 ± 1.2 and 3.4 ± 2.4 and that, in subsequent recurrences, the clinical AT is different to the original one in 79% of cases [21].

### 3.4. Predictive Factors of AT Occurrence

Several circumstances have been postulated to contribute to a more elevated risk of PAFAT: extensive ablation [21, 24, 26], incompletely ablated areas [15, 27, 62], PV reconnection [34, 38], early debut of atrial arrhythmias post AF ablation [18, 21, 35, 63], and previous long-lasting AF [17, 35]. Extensive ablation may increase the incidence of AT both directly, creating conduction barriers that eventually would define and stabilize a reentry circuit spatially separated from the ablated area [21, 64], and indirectly, enhancing the probability of conduction gaps [17], sometimes with complex tridimensional structure [65].

## 4. Types of PAFAT

### 4.1. Macroreentrant Circuits after AF Ablation (Figure 1)

#### 4.1.1. Frequency and Types

Macroreentrant (MR) ATs are considered the most frequent arrhythmias after AF ablation, comprising 57%–91% of the total. Nevertheless, some relevant studies have made clear that it may not always be so.Haïssagerre et al. [56] and Deisenhoffer et al. [15] reported that the so-called small-loop or localized reentry could be as frequent a mechanism as MR. Shah et al. [37], Ouyang et al. [38] and Gerstenfeld et al. [33] published data according to which focal or small-loop reentry was the most frequent mechanism (77%, 88%, and 100%, resp.). These striking differences could reflect not only, in the first instance, a renewed interest in a more detailed characterization of the frequently neglected “focal” atrial tachycardias [66–68], but also the possibility that more limited ablation strategies such as those used in these studies (PV segmental or circumferential isolation alone [33, 37, 38]) are not so liable to cause MR tachycardias. Perimitral atrial flutter is the most common type of MR circuit arising after AF ablation (39%–61%), closely followed by roof-related or peri-PV tachycardias (9%–61%), typical right atrial flutter (15%, although in some case series this was the most frequent arrhythmia [45, 52]), and other circuits involving the coronary sinus (5%–7%), the interatrial septum (10%–18%), or the anterior atrial wall [64] (3%) [15–21, 33, 37–39, 45, 52, 54, 56, 57, 62, 69]. It is noteworthy that complex dual-loop circuits are not rare (22%–55%) [16, 21].

#### 4.1.2. Identification and Mapping of MR Circuits

This mechanism is suspected when the activation sequence map yields results congruent with a sequentially continuous electrical activity accounting for at least 80%–90% of the tachycardia cycle length (CL). Activation sequence maps are usually obtained with tridimensional (3D) electroanatomic mapping systems in which every local activation time is represented as a color of a continuous spectrum, thus creating a visual representation of the sequence of activation. If the diameter of the circuit is ≥3 cm, the earliest activated area is close to the latest one (the typical “early meets late” or “head meets tail” aspect), and if the range of local activation times encompasses most of the CL, macroreentry is probable. The boundaries delimiting the circuit (such as sites harboring double potentials separated ≥50 ms, low-voltage areas characterized by voltages ≤0,05–1 mV, anatomical obstacles) are commonly included in the activation map representation in order to depict the course of the activation front with more anatomical accuracy. This method of creating an activation sequence map was the most frequently used in the studies previously cited. In spite of its widespread acceptation, activation maps can be sometimes misleading [56, 70] and lead to equivocal results. Typically, when the entire MR circuit is not accessible or some parts of the activation sequence cannot be registered with certainty [36, 71], the activation map of a MR circuit can be misinterpreted as an apparent centrifugal activation of the atria with a presumptive, but false, focal origin [36]. The limitations of the activation mapping are posed by a nonsystematic collection of points during which critical areas of the circuit [72] may be missed, the intrinsic difficulty of assigning an objectively exact activation time to a multicomponent or low-voltage fractionated electrogram, and the presence of multiple areas of slow conduction or conduction block resulting in an abnormally prolonged time to complete LA activation [73] in such a way that some atrial areas are activated very late. Some modifications of the conventional point-by-point construction of activation maps have been attempted in order to simplify the approach and gain accuracy. For example, the use of multielectrode catheters [72] or the elegant refinement of this technique reported by De Ponti et al. [74]. 

In addition, the documentation of a sequential atrial activation through the entire AT cycle length is insufficient to establish the existence of an atrial MR arrhytmia: the only unequivocal proof is the demonstration of fusion during entrainment or, what is even more compelling, return cycles after entrainment matching the tachycardia CL from at least two distant sites. In this respect, entrainment mapping with fusion affords the ultimate evidence of a reentrant mechanism irrespective of the “focal” appearance of the activation map [33, 36, 39, 71]. A detailed analysis of entrainment maps has not been carried out in most studies: in some cases entrainment techniques have been used [16, 19, 20, 28, 34, 75] only exceptionally, and in other cases entrainment has been used in a limited way just to confirm that certain sites of interest (particularly putative isthmuses candidate for ablation) belong to the circuit [17, 21, 22, 58, 62, 72]. On the other hand, entrainment mapping has been used by some investigators in a more comprehensive manner to determine the true spatial location of the circuit [15, 36, 57, 64]. Some authors have manifested a certain reluctance against an exhaustive use of entrainment mapping [76] not only because of its limitations (it is not always possible to demonstrate fusion, specially when reentrant circuits are small [37]) but also because there exist a potential risk of terminating the AT into sinus rhythm, a different AT or AF.

### 4.2. Focal Mechanisms (Figure 1) 

#### 4.2.1. Definition and Types

The identification of a focal AT [6] is based on two criteria: (a) centrifugal spread of the activation front in all directions from the site of earliest atrial activation [75], (b) range of activation duration less than the AT CL [16, 17], or in other words, sequential electrical activity accounting for less than 80%–90% of the CL [21, 22]. However, it is sometimes very difficult to discriminate between reentrant and focal mechanisms [36, 56], specially in a previously ablated atria. Due to this difficulty, a number of subordinate criteria have been proposed to suspect a focal origin [6, 33, 53, 57, 68]: (a) CL variations >10%, (b) discrete P-waves with clear isoelectric intervals between them, (c) inconsistent return cycles after entrainment pacing from several locations, (d) identical P wave and atrial activation sequence during pacing at the focus site and during the clinical AT, and (e) QS wave on the monopolar lead located on the origin of the AT. It must be noted that a focal origin of the electrical activation does not necessarily involve a “genuine” focal mechanism, such as enhanced automaticity or triggered activity. In this regard, it might be useful to remember that any confusion of terminology must be avoided: the term “focal AT” refers primarily to a pattern of concentric activation from a focus/source [6], the mechanism of which could indistinctly consist on (micro) reentry, abnormal automaticity, or triggered activity. In consequence, the use of the term “focal” as a synonym for *automatic or triggered mechanism* is confuse [6].

A particular subgroup of focal ATs [77] are characterized by (a) adenosine insensitivity (they present neither termination nor transient suppression), (b) low-amplitude potentials at the focus site, (c) long-duration electrograms (spanning a great portion of the cycle length) at the origin site, and (d) a response to overdrive pacing consistent with entrainment. All these features, considered together, strongly advocate reentry as the underlying mechanism. Sanders et al. [68] studied 27 tachycardias with a catheter specially designed for high-density mapping, establishing a localized reentry mechanism in 8 of them. The most relevant finding of this approach was the indisputable demonstration of sequential electrical activity during 95.2 ± 4.5% of the CL, together with prolonged fractionated electrograms at the sites of origin. Takahashi et al. [55] achieved similar results using conventional activation and entrainment mapping, but they also measured the size of the circuit (always <2 cm), the width of the isthmus (variable between <5 and 10 mm) and confirmed the spatial relationship of these small-loop reentry circuits to previously ablated areas. The close relationship between low-voltage zones (LVZs) or scar areas and the site of origin of the focal AT was reassured by Higa et al. [67] using noncontact mapping. Surrounding LVZs can sometimes configure a preferential exit channel from the AT focus [78]. These microreentry or small-loop reentry circuits fairly explain why Mohamed et al. [79] demonstrated that the closer to the AT focus the entrainment site is located, the shorter is the return cycle minus CL difference, a type of response that suggest a reentrant circuit when can be obtained consistently [80]. Finally, such sites harboring localized reentrant circuits have been directly proved or strongly proposed to be involved in the maintenance of AF [81, 82].

Deisenhofer et al. [15] have differentiated two types of small-loop reentry circuits (<3 cm) on the basis of a very reasonable argumentation: (a) small-loop reentry circuits related to gaps on previous ablation lines and (b) small-loop reentry circuits related to areas with markedly slow conduction, generally located in close proximity to previously ablated areas but not related to conduction gaps. Schematic small-loop reentry circuits resultant from the modification of a previous arrhythmogenic substrate by radiofrequency lesions were hypothesized by Merino in 2006 [73]. 

The other two focal mechanisms (automaticity and triggered activity) have not been so well defined in patients presenting PAFAT and would remain an exclusion diagnosis when MR or localized reentrant circuits could not be confirmed. Their typical responses to adenosine and overdrive pacing along with their typical clinical presentation as repetitive burst of tachycardia can serve as clues to suspect the diagnosis [77, 83].

#### 4.2.2. Frequency and Preferential Locations

In general, focal ATs constitute a minoritary group. For example, Deisenhofer et al. [15] did not report any focal ATs in their series, but it must be noted that 31% of the PAFATs they studied were too unstable for complete characterization. Gerstenfeld et al. [33], on the other hand, initially attributed a focal mechanism to all PAFATs they found after segmentary PVI. This assumption was somewhat doubtful because all these tachycardias manifested a fused intracardiac activation sequence during entrainment. On the basis of entrainment maneuvers the same authors published a second report [39] in which only one of five PAFATs had a presumable pure focal mechanism and the other four cases were small-loop reentries. These results underscore the capital importance of the differentiation between a *focal activation pattern* and a true *focal mechanism. *The first one habitually results from small-loop reentries or conduction barriers which can mask a MR mechanism [34, 73].

Rostock et al. [57], Chae et al. [21], and Mesas et al. [16] published a prevalence of focal AT which varies between 12–28% of the total PAFATs. The immense majority of these focal PAFATs were found near the PV antra in close relationship to previously ablated areas (41–100%) [16, 21, 57]. Apart from this preferential location, PAFATs with a confirmed or putative focal mechanism have been described as being scattered in many other places such as the coronary sinus (23%), interatrial septum (11-12%), LA roof and LA inferior wall [21, 57].

## 5. Electrophysiologic Characterization of PAFAT

A precise knowledge of the arrhythmogenic substrate as well as a great familiarity with the electrophysiologic properties of AT circuits is essential to identify and ablate their critical components.

### 5.1. Conduction Gaps

Conduction gaps are simply narrow parcels of surviving cardiac tissue still capable of effective electrical conduction which connect two zones otherwise separated by an interposed area of nonconducting tissue. This area of conduction block generally consists on scar tissue caused by radiofrequency or other modalities of energy delivered for ablation. Therefore, a gap is always a portion of cardiac muscle which has been either reversibly damaged by the ablation catheter, independently of the nature and intensity of the lesion (inflammation, edema, alteration of membrane properties [84]) or not damaged at all. Obviously, gaps can be already present immediately after the AF ablation procedure. These residual gaps are typical in atrial regions which are hardly accessible to radiofrequency energy for whatever reason: catheter instability, edema formation, tissue thickness that prevents the transmurality of the lesions, or tricky anatomical areas such as the ridge between the left upper pulmonary vein and the left atrial appendage [17, 85, 86]. More frequently, however, conduction gaps appear later as true “reconnections” [87] of the previously ablated areas.

#### 5.1.1. Relationship between Gaps and PAFAT

Despite multiple targeted radiofrequency applications during an AF ablation procedure, multiple or single gaps can persist in a significant number of patients (5–19%) [17, 88, 89]. These incomplete lines have been proved to be a strong promoting factor of AT [17, 27, 43]. The extreme examples of this cause-effect relationship are exemplified by some published cases of (a) acute organization of AF into a MR AT gaps located in ablation lines which have been deployed just a few minutes before [75] and (b) gap-dependent MR ATs which can be induced shortly after apparently complete PV circumferential isolation [60]. The reason most frequently adduced to explain the presence of conduction gaps is immediate or delayed conduction recovery. Certainly, this is the only acceptable hypothesis when PV isolation and bidirectional conduction block across other additional lines was reliably confirmed [16–18, 62, 89] previously. 

In turn, gap-related macroreentry is the most habitual type of MR PAFAT (65%–96%) [16, 21, 28, 58, 62, 89] and multiple gaps are habitually required [36, 71, 73]. In addition, a relevant proportion of focal or small-loop reentrant ATs have also been found spatially related to single (100% of focal AT reported by Mesas et al. [16] and Luik et al. [60]) or double gaps (35% of small-loop reentry reported by Deisenhofer et al. [15]). Pure PV tachycardias [73] after AF ablation as those published by Ouyang et al. [38] deserve a particular comment because they are a direct consequence of PV reconnections through gaps.

#### 5.1.2. Identification of Gaps

Conduction gaps are viable tissue surrounded by permanent lesions; in consequence, they are located in incompletely ablated areas which can be revealed by magnetic resonance image techniques due to the presence of preserved atrial muscle bundles. It has been demonstrated that up to a maximum of 20% of the surface of the tissue where radiofrequency has been applied (areas usually marked by lesion dots on the 3D navigation systems) may correspond to viable tissue (not affected by gadolinium late-enhancement) [90]. To date, however, the efficacy of this approach to detect very small bundles of viable atrial myocytes has not been validated. An indirect way to suspect the presence of conduction gaps in ablation lines is the simultaneous achievement of AT interruption and bidirectional block across the line using a single or a few focal radiofrequency applications [38]. A detailed activation map can offer a more direct proof of the participation of gaps in a MR circuit when the activation front traverses perpendicularly the place where a prior ablation line was created [21, 60]. The exact position of ablation lines relative to the AT circuit can be checked by side-by-side comparison of the AT activation map and the tridimensional reconstruction of the LA obtained during the initial AF ablation procedure, or with techniques of image integration implemented in modern 3D navigation systems, which allow for a more exact delineation of the spatial relationship between incomplete ablation lines and the circuit of the AT [91]. The most direct evidence which identifies a conduction gap is the presence of a single potential flanked by double potentials. The single potential (frequently fractionated) corresponds to the viable muscle and the double potentials to the ablation line lying at both sides of the gap [16].

#### 5.1.3. Conduction Properties of Gaps

Noncontact mapping technology has provided greatly detailed images of the activation wavefront propagation across an ablation line through a gap: the activation front narrows upon reaching the gap and widens again at the other side of the line [92]. However, electrical conduction through discontinuous ablation lesions is a complex phenomenon influenced by the width of the gap, its geometry, and possibly the time elapsed since the lesions were created. In this sense, certain experimental models suggests that there is a minimum width necessary for gaps to conduct (habitually 3 mm [93]). Besides, the gap size can modulate the conduction velocity of the wavefront crossing it, in such a way that the narrower the gap, the slower the conduction velocity [93]. As a result, small gaps are usually characterized by long-duration and very fractionated potentials small gaps usually showing [93–95]. The overall delay is not exceedingly great in comparison to normal tissue [94] and has been deemed to lack clinical relevance, insofar as small gaps are still capable of relatively fast conduction [38]. The solid results of Melby et al. [95] reaffirm both ideas: only very small gaps tend to manifest features of slow conduction (as a matter of fact, fragmented electrograms are a prominent distinctive mark of conduction gaps in the clinical setting [16, 38, 58]), and the overall conduction velocity impairment is small, allowing for effective conduction of very rapid paced rhythms and even AF.

On the other hand, conduction properties of gaps depend not only on their width but also on their geometrical configuration: angled or “L-” shaped gaps are much less likely to conduct than bifurcarted (“Y-” shaped) or straight gaps [65]. In general, sodium-channel blockers are effective in preventing gap-related electrical conduction in experimental models [65].

#### 5.1.4. Frequent Locations of Gaps

Rostock et al. [87] reported the distribution of sites of conduction recovery in roof lines and mitral isthmus lines: 66% of gaps in the mitral isthmus line were localized in the upper portion, close to the left inferior pulmonary vein and the left atrial appendage; 54% of gaps in the roof line were found in close relation to the right superior pulmonary vein ostium. Both preferential sites are the result of anatomical structures that condition a worse stability of the catheter and areas of greater wall thickness more difficult to ablate with transmural lesions. Similarly, Mesas et al. [16] found also a preferential location of gaps near the septum and the superior segment of the lateral left atrium, in both cases around the PV antra. Chang et al. [60] reported that the preferential location was another difficult place: the left atrial appendage ridge. However, Ouygang et al. [38] did not find any preferential distribution for 32 conduction gaps in circumferential lines around the PV ostia, in spite of the fact that gaps were approximately twice as frequent around the left PV.

### 5.2. Critical Isthmus

#### 5.2.1. Anatomic Description

An isthmus can be defined as the narrowest part of the circuit. This definition involves two elements which need a precise characterization. Firstly, the course of the activation front defined as entirely as possible by the middle of activation mapping, entrainment mapping, noncontact mapping or analogous techniques; secondly, the presence of conduction barriers delimiting the anatomic channel through which the activation front traverses. These boundaries can be normal anatomic structures, scar areas, areas of conduction block (double potentials) [96], or previously ablated areas. Jaïs et al. [97] published an interesting study in which they identified critical isthmuses on the basis of an exclusively anatomic concept: an *isthmus* is configured by two lateral barriers and a corridor of normally excitable atrial tissue between them. These authors also demonstrated that a line of ablation transecting these isthmi is an adequate therapy for most macroreentrant AT. Obviously, this approach can be resorted to if the ATs is not stable enough as to resist a detailed entrainment mapping [98] and the participation of the isthmus in the circuit can be demonstrated at least with activation mapping. However, Ouyang et al. [99] showed with conclusive data that isthmi are usually narrow and the typical electrograms registered within them are of very low voltage amplitude (generally <0.5 mV) and multicomponent or fragmented. As a result, isochronal and activation maps are specially limited to characterize them, because it is often really difficult to assign a reliable local activation time to such low-voltage and fragmented electrograms. Furthermore, purely anatomic isthmi are not always the feeblest part of the circuit. For example, the so-called *mitral isthmus* can be specially difficult to block bidirectionally [27], and this difficulty has led some authors to propose an alternative approach: the “anterior (or superior) line”. This line, traced from the mitral annulus to either superior PV, blocks the entire anterior aspect of the left atrium [100, 101].

#### 5.2.2. The Critical Isthmus from a Functional Point of View

De Ponti et al. [74] introduced a simple and elegant refinement of the conventional colour-coded activation map as a tool to help localize the zone of “diastolic” activation of the circuit. This strategy consists in a specific parameters setting of the 3D navigation system such that the location of the transition “purple-red” or “purple-white” (the traditional point where “head meets tail”) limit identifies the position of the diastolic isthmus, which, in comparison to the systolic isthmus, is more frequently characterized by low-amplitude potentials and slow conduction [74] and corresponds almost always to the most vulnerable part of the circuit. 

Entrainment mapping, in turn, is the most consistent method to determine whether a given point in the atrium belongs to the circuit or not [102, 103]. Isthmi can be defined with mere entrainment criteria [104] as those areas presenting concealed entrainment (defined as identical P-wave morphology and intra-atrial activation sequence during pacing and during AT), first postpacing interval equal to AT CL and a delay between the stimulus artifact and the elicited activation front of at least 40 msec. It seems clear, therefore, that activation mapping and entrainment mapping are complementary strategies [105] to localize the circuit and its course, boundaries, isthmi, slow-conduction areas, and, specially, sites where ablation has more chances to be effective, as Bogun et al. [104] studied in an excellent work only limited by the low number of cases included. Being it so, the true critical isthmus is not only a narrow corridor anatomically defined, but, above all, a site where the tachycardia is interrupted and rendered noninducible with the minimal number of radiofrequency applications. These sites hold some ancillary features which contribute to their identification: long activation times, split or fragmented electrograms, diastolic potentials and matching stimulus P-wave and electrogram P-wave intervals [104]. 

In the particular setting of PAFAT, isthmi can be constituted merely by gaps or by the interaction between ablated areas, preexisting scar areas and anatomic structures. Both an exclusive anatomical approach [15, 16, 18, 21, 22], and a more functional [17, 37, 61] characterization have been used to detect isthmi under those circumstances.

### 5.3. Low-Voltage Areas and Slow-Conduction Zones

#### 5.3.1. Preexisting and Iatrogenic Scar Zones and Areas of Slow Conduction

Whereas patchy fibrosis and increased concentration of type I collagen have been observed in patients with lone paroxismal AF [106], significant scar areas (characterized by very low voltage, ≤0,1 mV) have not been described in this group of patients [107]. Some authors have postulated the origin of abnormal atrial zones (LVZs, slow-conduction and scar areas) to be either totally iatrogenic or the result of the interaction between incomplete radiofrequency lesions and specific anatomic structures (ligament of Marshall, autonomic ganglia) [58, 108]. However, there are important cumulative data supporting the existence of these abnormal areas independently of radiofrequency lesions. Taclas et al. [90] noticed that late gadolinium enhancement was sometimes detectable in locations where radiofrequency had not been applied. Moreno-Reviriego et al. [109] demonstrated the presence of a dense scar (characterized by absence of capture at maximal paced impulse output) or low-voltage area in 10 of 16 patients with persistent/long lasting FA. Verma et al. [110] detected scar areas in 6% of AF patients and demonstrated the role of these areas as independent predictors of AF recurrences. Lo et al. [41] investigated the progressive decrease in the mean LA voltage and increase in the extension of low-voltage zones (subtracted the contribution of ablated areas) in patients with AF recurrences after PVI. Lin et al. [111] evaluated the role of areas characterized by functional conduction block and low voltage in delimiting slow-conduction isthmi as a common mechanism for right atrial flutter and fibrillation. Cummings et al. [34] appreciated that preexisting scar areas may act as an additional substrate for PAFAT because PV reisolation alone was less efficacious to prevent recurrences in patients with scar areas than in those without them. Jaïs et al. [64] found that some flutter circuits were constituted by areas of slow conduction distant from PV ostia and not targeted by prior ablation. Yoshida et al. [62] concluded that radiofrequency lesions cannot be directly linked to at least 30% of AT that appear late after AF ablation because these tachycardias were not adjacent to ablation sites. In addition, as previously discussed, there is still a lack of demonstrative evidence that slow conduction could be attributed to RF lesions. For example, in spite of the complex conduction properties manifested by partially ablated areas, slow conduction was ruled out by Chorro et al. [112] as the mechanism of the prolonged conduction time measured near radiofrequency lesions because it could be always explained by the conduction detour of the wavefront around the lesion. Besides, it remains without explanation why RF is specially (or exclusively) prone to beget slow conduction and iatrogenic arrhythmias in the LA and not in other substrates such as ventricular scars or RA flutters [73].

#### 5.3.2. Scars, Low-Voltage Zones, Slow-Conduction Areas, and Atrial Tachycardias

Independently of their origin, these abnormal areas may play a role in PAFAT for several reasons. First of all, LVZs are an integral part of the critical isthmus of most ATs. Secondly, LVZs and scars can simply make up the lateral boundaries delimiting a MR circuit, as is the case when a perimitral flutter is induced immediately after circumferential PVI [60] or after linear ablation at the roof, perhaps because the circuit is confined within these barriers and stabilized preventing short circuiting [21]. This constraining effect of natural and iatrogenic lateral barriers is most probably necessary for the maintenance of circuits unrelated to ablated zones [64]. There exist a more intricate cause-effect relation between areas of slow conduction and small-loop or localized reentrant AT, because when reentrant circuits are small in size, the phenomenon of slow electrical conduction acquires its most crucial relevance. Typically, small-loop reentrant circuits appear near previous ablation lesions and in places to which a special relevance for AF maintenance is commonly assigned, such as the PV antra [16, 21, 37] or the LAA opening [15, 46, 113]. The critical component of these circuits is a narrow isthmus showing typical low-amplitude fractionated electrograms which span a great part of the CL, indicating slow conduction [37]. In the most extreme examples [15] fractionated potentials lasting up to 140 ms and occupying 60% of the TCL or even the entirety of the CL can be registered [68, 81]. Deisenhoffer et al. [15] revealed something as important as frequently neglected: very slow-conduction areas are not located across ablation lines, on the contrary, they are simply adjacent to them. Consequently, it is reasonable to hypothesize that, if these slow-conduction areas existed before radiofrequency applications, they could have served as substrate for small and very rapidly rotating circuits implicated in the maintenance of AF. Posteriorly, RF lines could have modified the electrical properties of the circuit, for example, increasing its size and CL. The final result would be the creation of the mechanism of organized ATs as a consequence of the interaction between a previous abnormal substrate responsible for AF maintenance and radiofrequency lesions [73].

### 5.4. PV Reconnection and PV-Related Triggering Foci

PV-related foci of ectopic activity implicated in AF initiation and maintenance seem to play a relevant role in triggering organized PAFAT, although the participation of extra PV triggers should not be underestimated. 

As noted before, PVI is the only element of all the AF ablation techniques clearly associated with a reduced incidence of PAFAT. After AF ablation, the persistence of PVI is also fundamental for sinus rhythm maintenance. The most relevant piece of evidence was provided by the great proportion of patients in whom PV reconnection was demonstrated during the PAFAT study and ablation. Ouyang et al. [38] and Chun et al. [114] addressed the problem of PV reconnection in both case series of PAFAT after catheter-guided PVI and surgical Maze, respectively. Their results were incontrovertible: 80% of patients with AT recurrences after catheter ablation and 88% of patients with AT after Maze procedure presented PV reconnection. Similarly, according to a recent study by Sy et al. [115], in the group of patients requiring a second ablation procedure after PVI (48.3% presented recurrences in form of organized PAFAT), 82% of the PV were reconnected. Other authors [33, 34, 36, 39] have also indirectly proved the cause-effect relation between the presence of venoatrial reconnections and PAFAT. In addition, the contribution of ectopic activity arising from the PV to the initiation of organized AT is also proved by the capital importance of PV reisolation to reduce AT recurrences. For example, Cummings et al. [34] evaluated the effect of PV reisolation alone to treat LA flutters following a previous PVI procedure and obtained interesting results: 61% of patients remained free of arrhythmia recurrences off antiarrhythmics and an additional 21% on antiarrhythmics. The importance of abolishing the contribution of PV-related triggers was reported by Wazni et al. [116] as well. Patients with coexistent AF and typical right atrial flutter were included in this study and underwent PVI isolation without concomitant bidirectional cavotricuspid isthmus blockade, which was curative in most of them. Therefore, in the light of all these data PV reconnection remains the milestone of the pathologic process which ultimately leads to PAFAT occurrence, to such an extent that a certain dependence of organized AT on PV triggering foci has to be assumed. The question is whether this relationship is direct or indirect, or in other words, whether PV ectopics are able to initiate organized AT with or without an intermediate period of AF [116, 117]. Both possibilities are likely but it must be noted that, in a simulation study, Gong et al. [118] demonstrated that the atria are more vulnerable to premature beats arising from the PVs than from other locations and that the vulnerable window for atrial flutter/AF induction (the range of different pacing CL and extrastimuli coupling intervals) is markedly smaller for right atrial foci in comparison to PV foci. 

Two reasonable consequences can be drawn from the close relationship between PV reconnection and PAFAT. Firstly, PV reisolation should be considered the first procedural step when a PAFAT ablation is undertaken [14]. Secondly, all measures aiming at reducing the risk of PV reconnection have to be implemented. For example, it is commonly reckoned today that a mere anatomic atrial ablation guided by electroanatomic mapping systems is unreliable in achieving a complete PVI [24, 119]. Consequently, PVI should be assessed routinely by a circular-mapping-guided catheter approach, taking into account that critical areas such as the carina between ipsilateral veins have to be targeted for ablation almost always [120]. Finally, many laboratories have adopted the use of general anesthesia for AF ablation procedures in view of the lower probability of PV reconnection reported by Di Biase et al. [121] or have started to employ magnetic robotic navigation [122, 123]. This new technology has been suggested to improve the catheter stability or to simplify the PVI technique.

## 6. Results of Ablation

In the previous paragraphs we have enumerated the great number of strategies which have been published to localize and characterize the mechanism of PAFAT. All of them should be considered complementary approaches, and their use should be conditioned to the operator experience and to the particular requirements and peculiarities of each AT in an individualized manner. Basically, our experience and the published results of other authors invite to consider that activation mapping should always be complemented with entrainment maneouvres and that the tridimensional image support afforded by electroanatomic navigation systems is in general advisable to optimize the results of ablation. The algorithm proposed by Knecht et al. [14] and developed by Jaïs et al. [61] deserves the utmost attention because it provides accurate rules to elucidate the most frequent PAFAT mechanisms laying emphasis on simple and precise criteria: PVI reisolation is performed first and then focal, macro-reentrant, and small-loop reentrant mechanisms are systematically sought after in this order. 

In spite of the heterogeneity of techniques habitually resorted and the differences in the degree of mechanistic characterization of these arrhythmias, several authors have published convincing results (see Table 1) showing the success rate of PAFAT invasive treatment. The clinical PAFAT can be ablated almost always (70–100% of individual AT). Patel et al. [72] and Deisenhoffer et al. [15] reported a somewhat lower percentage of success (61% and 38%, resp.) which may be explained because most of their patients presented several different ATs. However, recurrences of new organized ATs are not rare (up to 21%–44%). Long-term sinus rhythm maintenance has not been conveniently explored by the studies summarized in Table 1 because the time of followup after the PAFAT treatment was not long enough in most cases.

## 7. Organized AT during AF Ablation

AF termination is considered by some authors a desiderate but not always attainable objective of AF ablation. The *distillation *[14] of AF into organized AT has been judged a reasonable way to modify the substrate responsible for AF maintenance. However, the extensive ablation this objective usually demands, together with the controversial results which have apparently disproved its efficacy in terms of effective prevention of AF recurrences [124], has precluded a more widespread acceptation of this opinion. At all events, organization of AF into AT during ablation is an interesting phenomenon, worthy of mention because it has given rise to new hypothesis about AF maintenance mechanisms. Some of these organized ATs are not spontaneous. For example, Chang et al. [28] induced organized AT in 16.3% of patients immediately after circumferential PVI, most of them (76%) were gap-related AT and macro-reentrant circuits around the mitral annulus or ipsilateral PVs were the most frequent. In fact, this inducibility guided strategy may be helpful to uncover gaps and latent AT circuits which could eventually acquire clinical relevance afterwards [18]. On the other hand, spontaneous ATs have much more interest from a mechanistic point of view because they could indicate the location of areas relevant for AF maintenance. Scharf et al. studied six cases of spontaneous conversion of AF into focal AT during left atrial ablation. All these ATs had a focal origin adjacent to ablated areas but unrelated to conduction gaps, and what is even more important, three of them had also an exact spatial correspondence with areas that seemed to anchor the fastest frequencies during AF (characterized by a CL during AF 30–40 ms shorter than the mean CL in adjacent zones). These focal sources of organized AT were correctly interpreted as slowed versions of very rapidly firing zones, the mechanism which had been probably modified by the ablation procedure. Only one of them manifested the typical behavior of an automatic focus but the mechanism was not investigated in great detail in the rest. A further and not less exciting insight into the hypothesis that AF and organized AT could be two sides of the same coin was provided by the study of Yoshida et al. [62] In this study the AF ablation strategy consisted in antral PVI and targeting of complex fractionated electrograms until AF converted to sinus rhythm or organized AT. There was a spectral component in the AF periodogram that matched the frequency of the resulting AT in 52% of patients who presented acute transformation of AF into AT. This proportion was higher (79%) when the AF preriodogram was evaluated just before conversion to AT. This correspondence between the frequency of the resulting AT and one of the components of the AF periodogram suggests that the AT circuit is already present during AF in some way or another, but hidden behind the fibrillatory proccess and surpassed by the higher-frequency components of the AF spectrum of frequencies. As for the mechanism of these resulting ATs, 94% were macro- or micro-reentry circuits, 70% of them located distant from the ablated areas. How crucial is the contribution of these lower-frequency drivers to the global fibrillatory phenomenon? It is evident that the lower-frequency components alone cannot result in fibrillatory activity once the higher-frequency components have been abolished. Nevertheless, it is not known whether the fibrillatory process requires not only the high-frequency drivers to be manifest but also the lower-frequency reentrant circuits to remain stable or what would happen if these subordinate components could be localized and ablated independently in the first instance. 

Organization of AF into AT during extensive CAFEs ablation occurs in 36% of cases of paroxysmal AF and in 50% of persistent AF [125]. Different mechanisms of AT in this context were described in pioneer works [46] and have been recently revisited by Nam et al. [53], who have reported the following results: 30% perimitral MR, 30% cavotricuspid isthmus-dependent flutter, 18% roof-dependent MR, 18% focal and 6% of unknown mechanism. These circuits are well-known, amenable to mapping, and commonly ablated in daily clinical practice, from whence comes the opinion that AF organization might be as good an outcome as termination [61].

## 8. Conclusions

The incidence of PAFAT is clinically relevant but depends on the ablation technique initially used to treat AF and on the existence of appropriate anatomic substrates, which sometimes exist before the ablation procedure. Both the mechanisms and elements constitutive of PAFAT circuits are well-known and have been repeatedly and consistently described. This fact, along with the high probability of success when these AT are targeted for ablation, and the low efficacy of antiarrhytmic drugs, implies that an invasive approach should be attempted if these AT became incessant, bad tolerated or do not disappear after a prudential observation period. However, it must be noted that the frequent coexistence of multiple mechanisms and several different types of PAFAT in the same patient determine the special complexity of these procedures, in which PV reisolation is the pivotal element when reconnection has occurred.

## Figures and Tables

**Figure 1:**
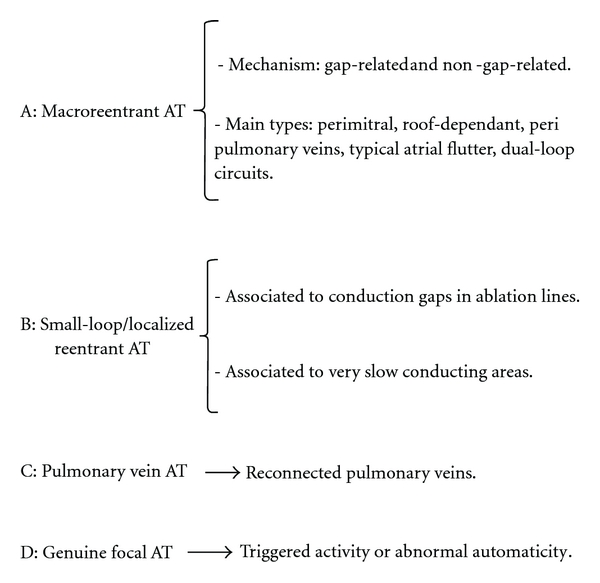


**Table 1:** 

Author and year	Number of patients	Number of ATs studied	ATs amenable to mapping	AT change during entrainment	AT change during RF	AT successfully ablated	Followup: persistence in SR	Followup: AT recurrence	Redo procedures for AT recurrence
Paponne et al. 2004 [17]	39	39	39/39 (100%)	Not reported	Not reported	39/39 (100%)	39/39 (100%)	6/39 (15%) early after ablation	0%
Kobza et al. 2004 [20]	10	20	19/20 (95%)	Not reported	Not reported	16/19 (84%)	Not reported	1/10 (10%)	Not reported
Mesas et al. 2004 [16]	13	14	12/14 (86%)	Not reported	1/14 (7%)	13/14 (93%)	11/13 (85%)	1/13 (8%)	1/13 (8%)
Gerstenfeld et al. 2004 [33]	10	10	9/10 (90%)	1/10 (10%)	0%	9/10 (90%)	9/10 (90%)	0%	0%
Chugh et al. 2005 [18]	28	>28 (30 reported)	28/30 (93%)	1/30 (3%): original AT could not be reinduced	Not reported	Success in 22/28 patients (79%)	18/22 (82%)	3/22 (14%)	3/22 (14%) patients after a successful procedure
Ouyang et al. 2005 [38]	21	17	15/17 (88%)	Not reported	Not reported	14/15 (93%)	21/21 (100%)	0%	0%
Cummings et al. 2005 [34]	23	Only PV reisolation	PV reisolation only	Not reported	Not reported	100% reisolation success	82% of patients	9% of patients	9% of patients
Jaïs et al. 2006 [64]	14	14	Only a specific type of AT	Not reported	Not reported	14/14 (100%)	11/14 (79%)	3/14 (21%)	2/14 (14%)
Daoud et al. 2006 [19]	9	17	13/17 (76%)	Not reported	Not reported	16/17 (94%)	100% of patients with successful procedure	0%	0%
Chae et al. 2007 [21]	78	155	155/155 (100%)	Not reported	Not reported	134/155 (86%)	60/78 (77%)	18/66 (27%) acute success	14/66 (21%)
Patel et al. 2008 [72]	17	41	33/41 (80%)	7/33 (21%): original AT could not be reinduced	Not reported	25/41 (61%)	13/17(76%)	4/17 (24%)	2/17 (12%)
Satomi et al. 2008 [36]	8	8	Only a specific type of AT	Not reported	Not reported	8/8 (100%)	7/8 (88%)	0%	0%
Takahashi et al. 2008 [55]	9	Multiple (>15) AT	All except one	Not reported	4/9 (44%), all localized reentries	All except 3	8/9 (89%)	1/9 (11%)	None
Lim et al. 2008 [89]	18	≥23	20 (at least 3 were not stable enough to be mapped)	Not reported	Not reported	20/20 (100%)	Not clearly reported, 79% including AF patients	Not reported	Not reported
Deisenhoffer et al. 2009 [15]	16	55	38/55 (69%)	Not reported	Not reported	23/55 (42%)	6/16 (38%)	7/16 (44%)	5/16 (31%)
Chang et al. 2009 [28]	26	48	48/48 (100%)	Not reported	5/48 (10%)	48/48 (100%)	20-21/26 (79%)	1/26 (19%)	1/26 (19%)
Rostock et al. 2010 [57]	61	133	132/133 (99%)	5/132 (3,8%)	45/61 initial AT (74%)	124/133 (93%)	50/61 (82%)	7/61 (11,5%)	5/61 (8%)
